# The Modulation of Nrf-2/HO-1 Signaling Axis by *Carthamus tinctorius* L. Alleviates Vascular Inflammation in Human Umbilical Vein Endothelial Cells

**DOI:** 10.3390/plants10122795

**Published:** 2021-12-17

**Authors:** Yun Jung Lee, Yong Pyo Lee, Chang Seob Seo, Eun Sik Choi, Byung Hyuk Han, Jung Joo Yoon, Se Hoon Jang, Chae Ghang Jeong, Yeun Ja Mun, Dae Gill Kang, Ho Sub Lee

**Affiliations:** 1 Hanbang Cardio-Renal Syndrome Research Center, Wonkwang University, 460, Iksan-daero, Iksan 54538, Korea; shrons@wku.ac.kr (Y.J.L.); arum0924@naver.com (B.H.H.); mora16@naver.com (J.J.Y.); wkdtpgns321@naver.com (S.H.J.); 2 Professional Graduate School of Korean Medicine, College of Korean Medicine, Wonkwang University, 460, Iksan-daero, Iksan 54538, Korea; dmstlr5421@gmail.com (E.S.C.); yjmun@wku.ac.kr (Y.J.M.); 3 Division of Infectious Disease Diagnosis Control, Honam Regional Center for Disease Control and Prevention, 103 Sangmusimin-ro, Seo-gu, Gwangju 62298, Korea; sendtosky@nate.com; 4 Herbal Medicine Research Division, Korea Institute of Oriental Medicine, 1672 Yuseong-daero, Yuseong-gu, Daejeon 34054, Korea; csseo0914@kiom.re.kr; 5 North London Collegiate School Jeju, 33 Global Edu-ro, Seogwipo, Jeju 63644, Korea; nalago070@gmail.com

**Keywords:** *Carthamus tinctorius*, HUVEC, vascular inflammation, HO-1, Nrf-2, NF-κB

## Abstract

*Carthamus tinctorius* L., known as safflower, has been used in traditional treatment for cardiovascular, cerebrovascular, and diabetic vascular complications. We proposed to investigate how the ethanol extract of *Carthamus tinctorius* L. (ECT) can be used ethnopharmacologically and alleviate vascular inflammatory processes under cytokine stimulation in human vascular endothelial cells. Using the optimized HPLC method, six markers were simultaneously analyzed for quality control of ECT. Pretreatment with ECT (10–100 μg/mL) significantly reduced the increase of leukocyte adhesion to HUVEC by TNF-α in a dose-dependent manner. Cell adhesion molecules (CAMs) such as intracellular adhesion molecule-1 (ICAM-1), vascular cell adhesion molecule-1 (VCAM-1), and endothelial cell selectin (E-selectin) are decreased by ECT. In addition, ECT significantly suppressed TNF-α-induced oxidative stress referring to reactive oxygen species (ROS) production. p65 NF-κB nuclear translocation and its activation were inhibited by ECT. Furthermore, pretreatment of ECT increased the HO-1 expression, and nuclear translocation of Nrf-2. These data suggest the potential role of ECT as a beneficial therapeutic herb in vascular inflammation via ROS/NF-kB pathway and the regulation of Nrf-2/HO-1 signaling axis is involved in its vascular protection. Thus, further study will be needed to clarify which compound is dominant for protection of vascular diseases.

## 1. Introduction

Atherosclerotic cardiovascular disease is the leading cause of death globally according to a report of published by Statistics WTO, and cerebrovascular disease and cardiovascular disease were the major causes of mortality. Atherosclerosis is a pathologic process caused by oxidative stress, chronic vascular dysfunction, and vascular inflammation. Once vascular endothelium is activated, leukocytes attach to vascular endothelium, subsequently migrate into the vessel wall. Infiltrated leukocytes can be differentiated into macrophages and release tumor necrosis factor-α (TNF-α) and consequently crucial risk factor for atherosclerosis because inflammation is closely regulated by signaling processes in vessel [1,2]. In endothelium, cell adhesion molecules play crucial roles in these events. Intracellular adhesion molecule-1 (CD54, ICAM-1) is an 85–110 kDa integral membrane glycoprotein. Vascular cell adhesion molecule-1 (VCAM-1, CD106), 110 kDa transmembrane glycoprotein, is constitutively expressed in vascular endothelial cells and upregulated by cytokine stimulation [3,4]. Furthermore, chemokines such as monocyte chemoattractant protein-1 (MCP-1) have been reported to stimulate non-leukocytes to secrete cytokines and CAMs [5].

Intracellular reactive oxygen species (ROS) play crucial roles in several physiological processes; they impair proteins, lipids, and DNA by inducing oxidative stress due to their highly reactive nature [6]. Vascular cytokines induce the production of ROS and activate redox signaling pathways, resulting in atherosclerosis [7]. They also stimulate p65 nuclear factor-κB (NF-κB), a transcription factor of pro-inflammatory genes, such as CAMs [8].

Heme oxygenase-1 (HMOX1, HO-1) is a target gene of nuclear factor erythroid 2-related factor 2 (Nrf2), which has been shown to protect against a variety of pathologies including sepsis, hypertension, and atherosclerosis [9]. HO-1 is further responsible for catalyzing the disintegration of heme into the antioxidant biliverdin, which is an anti-inflammatory agent, carbon monoxide, and iron [10]. Metabolites such as ferric iron, CO, and bilirubin are known to have antioxidative, anti-inflammatory, and anti-atherogenic properties [11]. The HO-1 deficiency model is related to endothelial cell injury, weakness, stress, ischemia, and growth retardation. In contrast, experimental HO-1 gene delivery has been shown to alleviate atherosclerosis [12], vascular neointima formation [13], ischemic heart injury [14], and vascular dysfunction [15].

*Carthamus tinctorius* L. (known as Safflower) belongs to the Compositae family and is distributed in South Asia, China, Iran, and Egypt [16,17]. The flowers of *C. tinctorius* are traditionally used to treat cardiovascular, cerebrovascular, gynecological, and diabetic vascular complications [18]. It is regarded to have beneficial coagulant and antioxidative effects, as well as beneficial effects against cancer and cardiovascular disease [19]. However, the specific effect of *C. tinctorius* flowers on the inhibition of vascular inflammation remains unclear. In the present study, we tested the hypothesis that the ethanol extract of the flowers of *C. tinctorius* (ECT) could decrease the levels of vascular inflammation markers in response to TNF-α, upregulate HO-1, and thus, protect against atherosclerosis. Therefore, the present study aimed to confirm the anti-vascular inflammatory effect of ECT and the molecular mechanisms underlying its action in human umbilical vein endothelial cells (HUVECs).

## 2. Materials and Methods

### 2.1. Preparation of ECT

Flowers of *C. tinctorius* L. (Compositae) were obtained from the Herbal Medicine Cooperative Association (Iksan, Korea) in 2018 and specimen voucher number was HBN321-01. The dried flowers of *C. tinctorius* (200 g) was soaked in 2 L ethanol at room temperature for 7 days, then concentrated using a rotary evaporator (Eyela, Tokyo, Japan). The concentrated extract (50 g) was lyophilized using a freeze-drier (PVTFD10RS, IlShinBioBase, Yangju, Korea) and dissolved in dimethyl sulfoxide (DMSO, <0.1%) for experiments.

### 2.2. Chemicals and Reagents for HPLC Analysis

**Figure 1:**
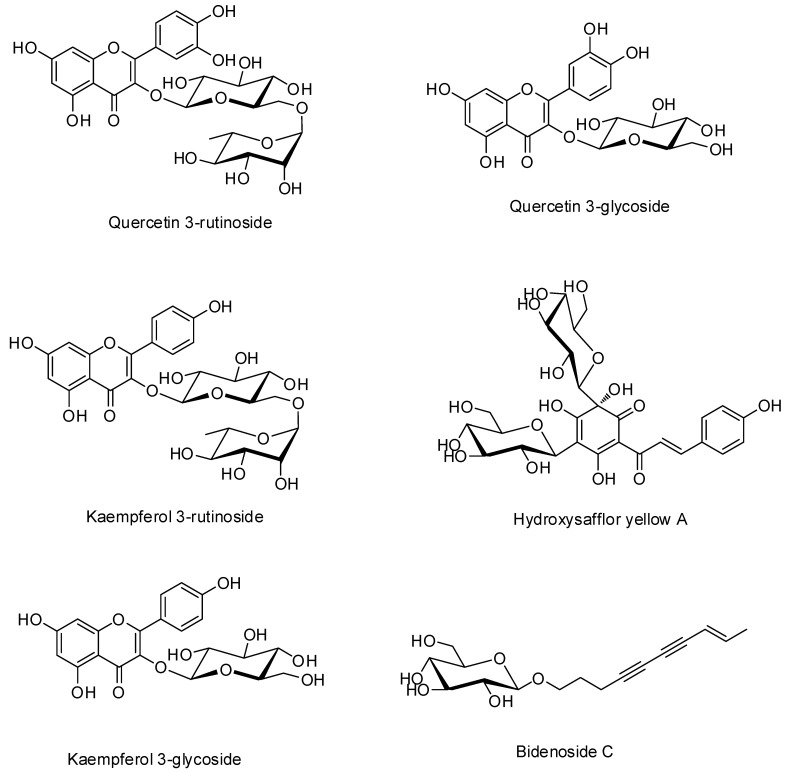
Chemical structures of six marker compounds in flowers of *C. tinctorius*. Four flavonoids, kaempferol 3-glucoside, kaempferol 3-rutinoside, quercetin 3-glucoside, and quercetin 3-rutinoside were included. One miscellaneous, bidenoside C, and one chalcones, hydroxysafflor yellow A were also included in flowers of *C. tinctorius*.

Four flavonoids, kaempferol 3-glucoside (C21H20O11, MW 448.38, purity 99.5%, Cat No. DR10611) and kaempferol 3-rutinoside (C27H30O15, MW 594.52, purity 98.2%, Cat No. DR11211) were purchased from Shanghai Sunny Biotech (Shanghai, China), and quercetin 3-glucoside (C21H20O12, MW 464.09, purity 98.2%, Cat No. CFN98753) was purchased from ChemFaces Biochemicals (Wuhan, China), and quercetin 3-rutinoside (C27H30O16, MW 610.517, purity 97.2%, Cat No. PHL89270) was purchased from Merck (Darmstadt, Germany). One miscellaneous, bidenoside C (C16H22O6, MW 310.4, Cat No. purity 99.8%, CFN95266) and one chalcones, hydroxysafflor yellow A (C9H10O5, MW 612.5, purity 99.7%, Cat No. CFN99950) were obtained from ChemFaces Biochemicals (Wuhan, China). Figure 1 illustrates the chemical structures of these reference standard compounds. High-performance liquid chromatography (HPLC)-grade solvents (methanol, acetonitrile, and water) and reagent, formic acid (≥99.7%) for HPLC qualitative/quantitative analysis were obtained from J. T. Baker (Phillipsburg, NJ, USA) and Thermo Fisher Scientific (San Jose, CA, USA), respectively.

### 2.3. HPLC Analysis of Six Marker Analytes in Flowers of C. tinctorius

Qualitative/quantitative for quality control of flowers of *C. tinctorius* was performed using a Shimadzu Prominence LC-20A Series (Kyoto, Japan) by controlled LCSolution software (Version 1.24, SP1). The mobile phase was comprised of 0.1% (*v*/*v*) aqueous formic acid (A), and 0.1% (*v*/*v*) formic acid in acetonitrile (B), and flowed under gradient elution conditions: 0–30 min, 20–50% B; 30–35 min, 50% B; 35–40 min, 50–20% B; and 40–50 min, 20% B. Quantification was measured at 265 nm for bidenoside C, 345 nm for kaempferol 3-rutinoside and kaempferol 3-glucoside, 355 nm for quercetin 3-rutinoside and quercetin 3-glucoside, and 400 nm for hydroxysafflor yellow A.

### 2.4. Cell Cultures

HUVEC and human monocytic leukemia HL-60 were obtained from American Type Culture Collection (ATCC; Manassas, VA, USA), and maintained at 37 °C in a humidified 5% CO_2_ atmosphere. Culture medium of HUVEC was Endothelial cell growth medium with 20% FBS, and that of HL-60 was RPMI 1640 with 10% FBS. All medium contained 100 U/mL penicillin, 100 μg/mL streptomycin. HUVECs were used between passages 4 and 5. To obtain confluent monolayers, HUVECs were seeded at a density of 2 × 10^4^ cells/cm^2^, and were used 36 h after incubation. The medium was changed at 24 h after seeding cell.

### 2.5. Enzyme-Linked Immunsorbent Assay

HUVEC were probed with mouse anti-human ICAM-1 (SC-8439, Santa Cruz, CA, USA), VCAM-1 (SC-13160, Santa Cruz, CA, USA), or E-selectin (SC-14011, Santa Cruz, CA, USA), antibodies at dilution (1:1000), in 1% BSA, in 2 h room temperature, after the cells were fixed by 1% paraformaldehyde. The cells were then incubated with the horseradish peroxidase-conjugated secondary antibody. Finally, a peroxidase substrate solution was added and measured at the 490 nm absorbance by a microplate reader (BioRad 3550, Hercules, CA, USA).

### 2.6. Western Blot Analysis

Cell lysates (protein of 40 μg) were run on 10% SDS-polyacrylamide gel electrophoresis, subsequently transferred to nitrocellulose membrane. Blots were blocked with 5% BSA and then exposed to the appropriate primary antibody in dilutions (1:1000) suggested from the commercial supplier. Primary antibodies were probed with horseradish peroxidase conjugated goat anti-rabbit-IgG (SC-2004, Santa Cruz, CA, USA) or anti-mouse-IgG (SC-2005, Santa Cruz, CA, USA). Visualization was performed with enhanced chemiluminescence (RPN2209, Amersham, Buckinghamshire, UK). Capturing image was achieved by ChemiDoc image analyzer (Bio-Rad, Hercules, CA, USA).

### 2.7. Total mRNA Preparation and Reverse Transcription-Polymerase Chain Reaction (RT-PCR)

Isolation of total mRNA was conducted using Trizol reagent (#15596026, Invitrogen). cDNA synthesis was performed by reverse transcription at 37 °C for 60 min, 94 °C for 5 min, respectively (#EZ105S, Enzynomics, Daejeon, Korea). The specific sequences of primers are shown in Table 1 [20]. PCR pre-mix was composed of mixture of template cDNA and 50 nM primers, reference to the manufacturer instruction (#25161, Intron, Korea). The amplification steps were followed by 45 cycles at 94 °C, 20 s; 60 °C, 20 s; 72 °C, 30 s. The final PCR products were determined by ChemiDoc image analyzer.

### 2.8. HL-60 Monocyte and HUVEC Adhesion Analysis

HUVEC were cultured to confluence in 24-well plates. HL-60 were labeled with 10 μM BCECF-AM (#B1170, ThermoFisher, USA) at 37 °C and added 2.5 × 105 cells to the HUVEC and then incubated for 1 h. Non-adhesive HL-60 were removed from the plate and HL-60 attached to HUVEC were observed by fluorescence microscope (Axiovision 4, Zeiss, Germany). The fluorescent intensity was measured at 485/585 nm (F-2500, Hitachi, Tokyo, Japan). The adhesion was represented in terms of change (%) compared with the control.

### 2.9. Intracellular ROS Production Assay

The confluent HUVEC were pretreated with ECT and incubated with 20 μM CM-H2DCFDA (#C6827, ThermoFisher, Waltham, MA, USA) and then treated with TNF-α for 6 h. The measurement of fluorescence intensity was obtained by spectrofluorometer and captured under a fluorescence microscope.

### 2.10. NF-κB Activation Assay

The extraction of nuclear proteins from HUVEC was carried out utilizing the nuclear and cytoplasmic protein extraction kit (#78833, ThermoFisher, Waltham, MA, USA). The levels of p65 NF-κB in nuclear extracts were determined by TransAM NF-κB kit according to the supplier’s instructions (#40096, Active Motif, Carlsbad, CA, USA).

### 2.11. Immunofluorescence Microscopy

HUVECs on Lab-Tek II chamber slides were fixed in 4% paraformaldehyde and permeabilized by 0.1% Triton X-100. Cells were probed with NF-κB p65 antibody (SC-8008, Santa Cruz, CA, USA) followed by fluorescein isothiocyanate (FITC)-labeled secondary antibody (a11001, Santa Cruz, CA, USA), and DAPI, respectively (Santa Cruz, CA, USA). The cells were finally mounted with Dako Fluorescent mounting medium (#CS70330-2, Agilent Technologies, Santa Clara, CA, USA) onto glass slides, and analyzed under a fluorescence microscope (Axiovision 4, Zeiss, Germany).

### 2.12. Statistical Analysis

The results were displayed as mean ± standard error (S.E.) and were analyzed using one-way ANOVA; Dunnett’s test or Student’s *t*-test using version 10 Sigma Plot software to determine any significant differences. *p* < 0.05 was represented as statistical significance.

## 3. Results

### 3.1. Quantification of Six Marker Compounds in ECT

Using the optimized HPLC method, six marker compounds (quercetin 3-rutinoside, quercetin 3-glycoside, kaempferol 3-rutinoside, hydroxysafflor yellow A, kaempferol 3-glycoside, and bidenoside C) were simultaneously analyzed for quality control of *C. tinctorius*. Retention times of these analytes were 9.47, 11.09, 11.83, 12.56, 13.88, and 21.24 min, respectively. Linear range, regression equation, coefficient of determination (r2), limit of detection (LOD), and limit of quantification (LOQ) for verifying the appropriateness of the established method are shown in Table 2. As a result of quantifying the marker compounds in flowers of *C. tinctorius* using this HPLC method, it was detected as 0.02–1.59 mg/g (Table 3).

### 3.2. Effect of ECT on TNF-α-Induced CAMs Expression in HUVEC

In order to estimate cytotoxic effects of ECT on HUVEC, MTT assay was performed. ECT did not show cytotoxic effects up to 100 μg/mL (>90% cell viability). Therefore, 100 μg/mL of ECT was used as the maximum dose in the present study (data not shown).

**Figure 2:**
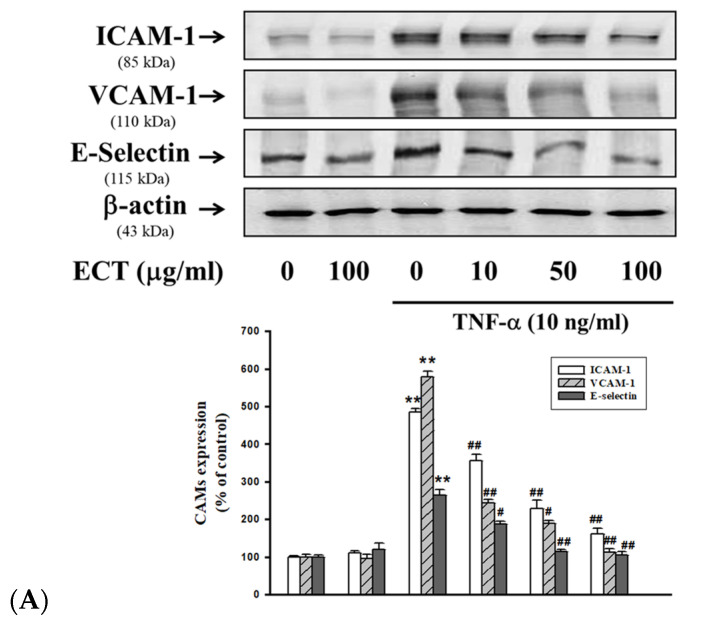

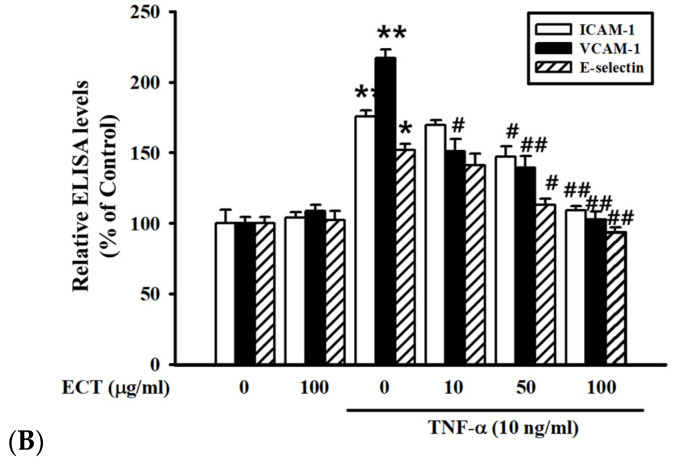
Effects of ECT on TNF-α-stimulated expression of cell adhesion molecules in HUVECs. Western blots (**A**) and cell surface expressions (**B**) of ICAM-1, VCAM-1, and E-selectin were analyzed according to Materials and Methods. Cells underwent 24 h 10 ng/mL TNF-α treatment in the absence or presence of 10–100 μg/mL ECT for 30 min. The blots are representative of three independents. Bars indicate the means ± SEM of three independent experiments. * *p* < 0.05, ** *p* < 0.01 vs. non-treated control; # *p* < 0.05, ## *p* < 0.01 vs. TNF-α.

**Figure 3:**
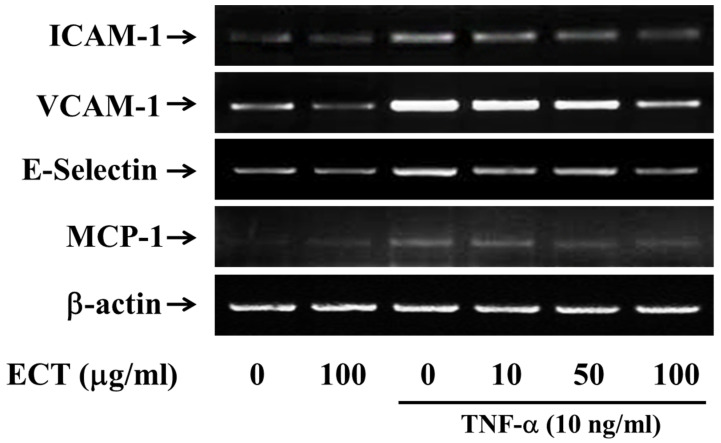
Effects of ECT on TNF-α-stimulated ICAM-1, VCAM-1, E-selectin, and MCP-1 mRNA expressions. Cells were treated with 10 ng/mL TNF-α for 24 h with/without 10–100 μg/mL ECT for 30 min and then were analyzed by qRT-PCR. The blots are representative of three independents.

We first determined the inhibitory effect of ECT on the CAMs expression on TNF-α-stimulated HUVEC by Western blotting and ELISA assay. Protein expression levels of ICAM-1, VCAM-1, and E-selectin were increased by TNF-α treatment and these effects of TNF-α were significantly suppressed by 10–100 µg/mL ECT (Figure 2A,B). In addition, mRNA expression levels of ICAM-1, VCAM-1, and E-selectin were also reduced by ECT in TNF-α-stimulated cells (Figure 3). These data indicate that ECT can improve to pro-inflammation process by regulating CAMs in vascular endothelial cells.

### 3.3. Effect of ECT on Leukocyte Adhesion to Vascular Endothelial Cells

**Figure 4:**
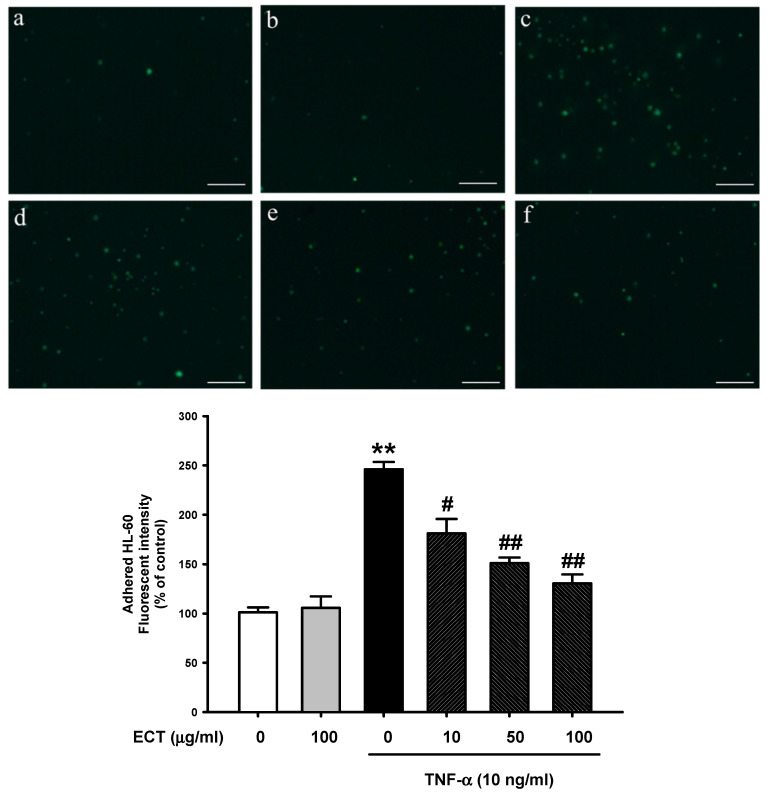
Effects of ECT on TNF-α-stimulated adhesion of HL-60 to HUVEC. Fluorescence of the adhered HL-60 was captured by a fluorescent microscope (200×) and quantified by microplate reader. Scalebars shows 100 μm. (**a**), control; (**b**), 100 μg/mL ECT; (**c**), TNF-α; (**d**), 10 μg/mL ECT + TNF-α; (**e**), 50 μg/mL ECT + TNF-α; (**f**), 100 μg/mL ECT + TNF-α. Bars represent the means ± SEM of three independent experiments. ** *p* < 0.01 vs. non-treated control; # *p* < 0.05, ## *p* < 0.01 vs. TNF-α.

Based on the above results, the effect of ECT on adhesion of HL-60 under cytokine stimulation was confirmed. HUVEC was pretreated with ECT (10–100 μg/mL), exposed to TNF-α and added fluorescent HL-60. As shown in Figure 4, the number of HL-60 adhesive to HUVEC was markedly increased by TNF-α. However, pretreatment with ECT significantly reduced the adhesion of HL-60 to HUVEC in a dose-dependent manner. The untreated HUVEC showed minimal binding to HL-60. These data suggest that ECT inhibited infiltration of macrophages, found in early atherosclerotic process.

### 3.4. Effect of ECT on TNF-α-Induced ROS/NF-κB Pathway

**Figure 5:**
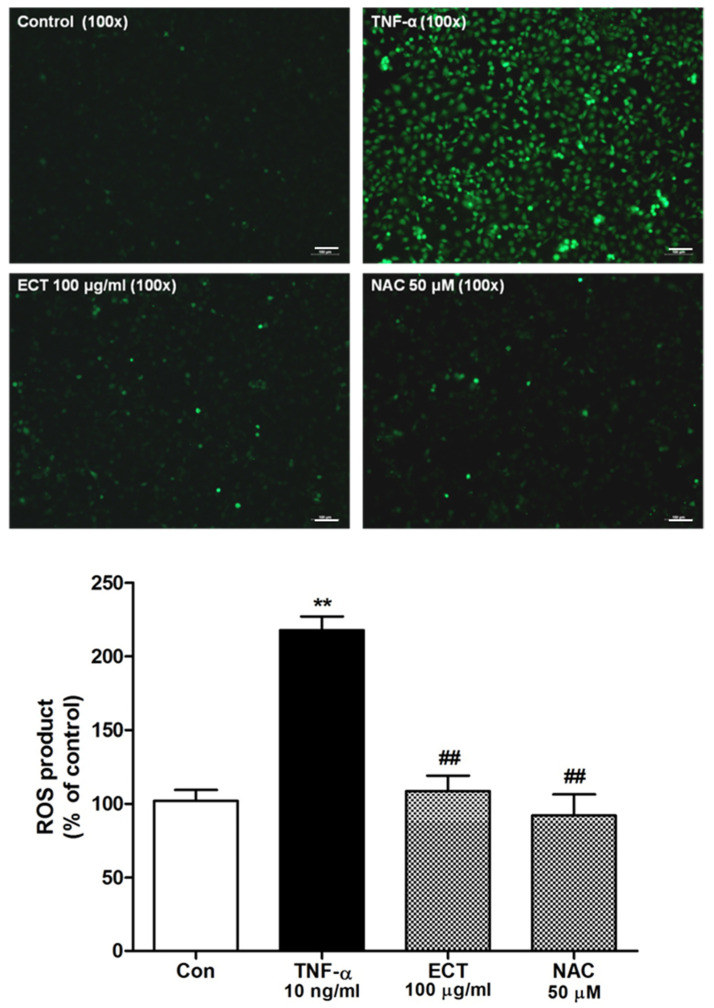
Effects of ECT on TNF-α-stimulated intracellular reactive oxygen species (ROS) generation. Cells were treated with TNF-α with/without 100 μg/mL ECT and then incubated with H_2_DCFDA. ROS generation was measured by a fluorescent microscope (200×) and microplate reader. Scalebars shows 100 μm. The lower bars represent the means ± SEM of more than three independent experiments. ** *p* < 0.01 vs. non-treated control; ## *p* < 0.01 vs. TNF-α.

Oxidative stress is a crucial factor of atherosclerosis pathophysiology and TNF-α induces ROS production in vascular tissue. HUVEC were labeled with a cell-permeable fluorescent probe, CM-H_2_DCFDA. As shown in Figure 5, intracellular ROS generation was markedly increased by TNF-α stimulation compared to untreated cell (Control). However, pretreatment with 100 μg/mL ECT significantly reduced ROS production by TNF-α; its effect similarly appeared like that of ROS scavenger *N*-acetyl-l-cysteine (NAC).

**Figure 6:**
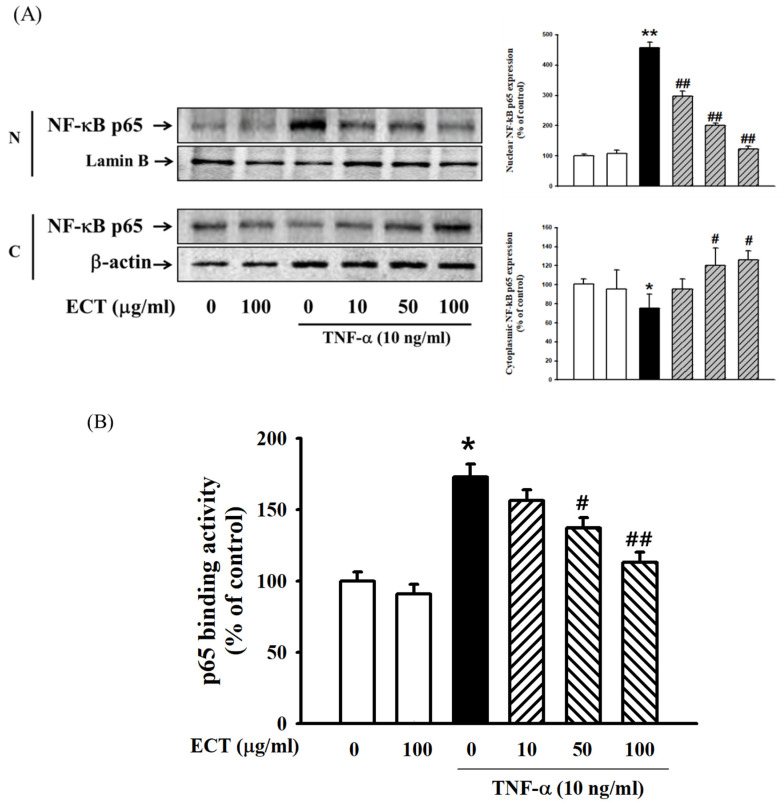

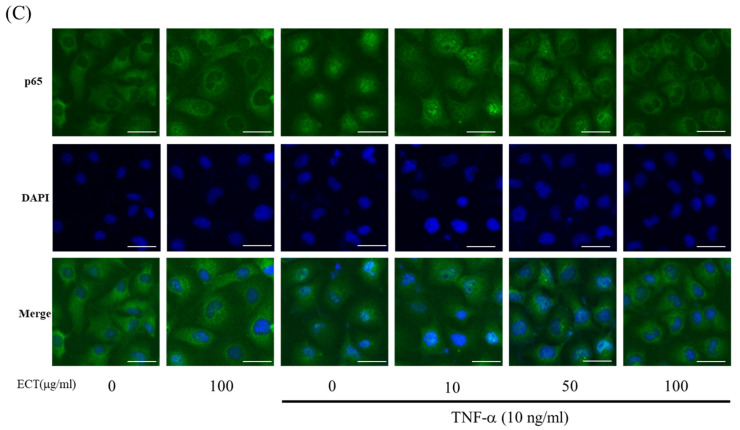
Effects of ECT on TNF-α-stimulated p65 NF-κB nuclear translocation. (**A**) Cells were pre-treated with TNF-α with/without 10, 50, 100 μg/mL ECT. Nuclear and cytosolic NF-κB p65 protein was detected by Western blot, respectively. (**B**) Nuclear extracts were evaluated by ELISA for the presence of p65 NF-κB. (**C**) p65 NF-κB was detected by Western blot and immunofluorescence (NF-κB, green; Nucleus, blue). Scale bars = 75 µm, original magnification is 400×. Magnification is 400×. Each bar represents the means ± SEM of three independent experiments. * *p* < 0.05, ** *p* < 0.01 vs. non-treated control; # *p* < 0.05, ## *p* < 0.01 vs. TNF-α.

Next, p65 NF-κB translocation from the cytoplasm to the nucleus was analyzed by Western blot. After treatment with TNF-α, the level of p65 NF-κB increased in the nucleus, while its level was decreased in the cytoplasm. ECT markedly inhibited TNF-α-induced nuclear translocation of p65 NF-κB (Figure 6A). Concomitantly, ECT suppressed the NF-κB activity that binds to DNA in TNF-α-stimulated HUVEC (Figure 6B,C). These data suggest that ECT suppress ROS/NF-κB pathway under TNF-α stimulation.

### 3.5. Involvement of HO-1/Nrf2 Pathway in ECT Protective Effect against Vascular Inflammation

**Figure 7:**
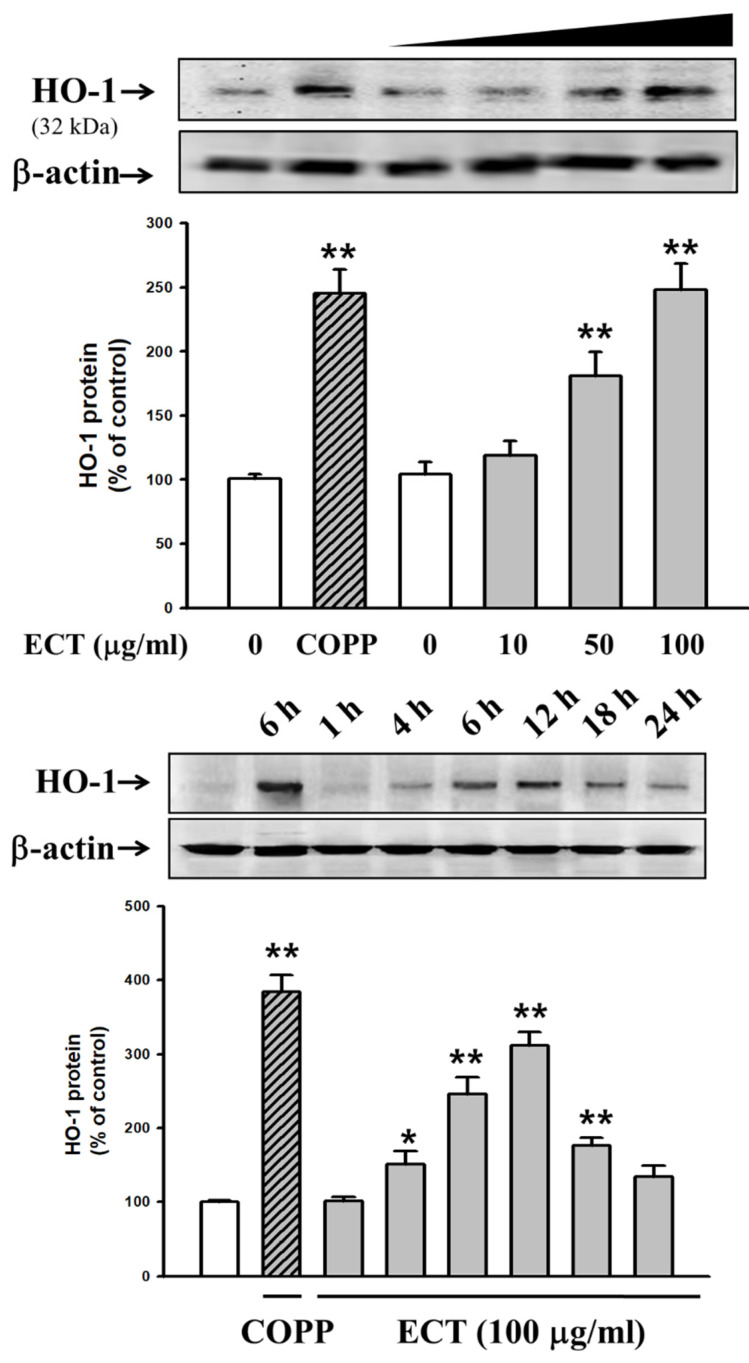
Effects of ECT on HO-1 induction. HUVECs were treated with 5 μM COPP or ECT (dose 10–100 μg/mL, time 1–24 h, respectively) as described in Methods. Each bar represents the means ± SEM of three independent experiments. * *p* < 0.05, ** *p* < 0.01 vs. control group.

**Figure 8:**
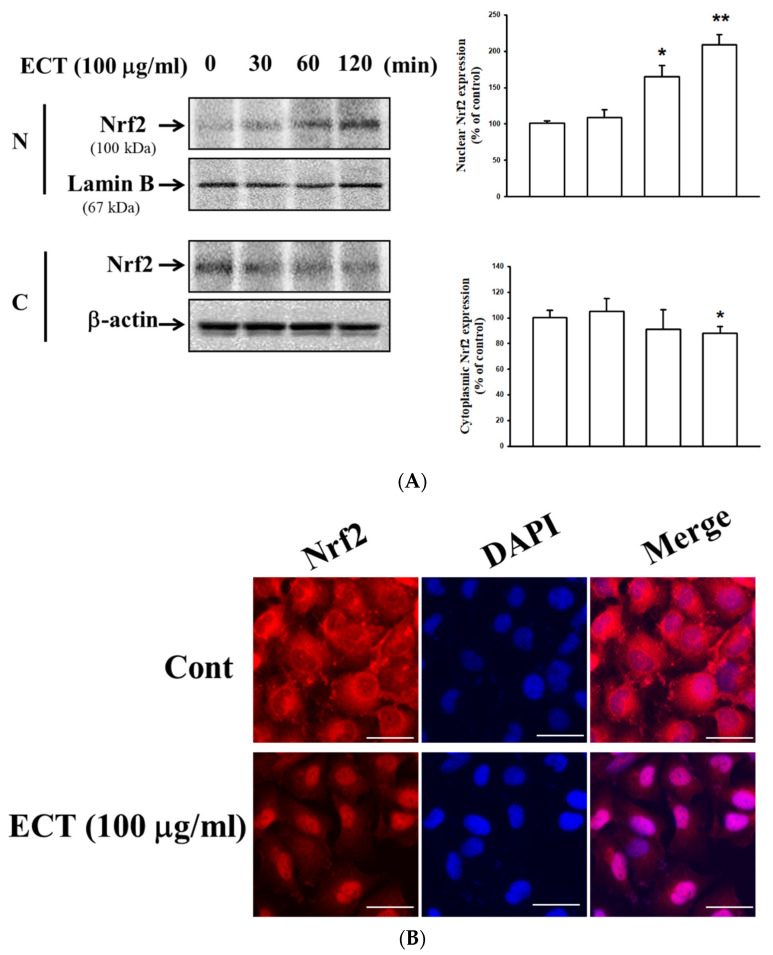
Effects of ECT on Nrf2 nuclear translocation. HUVECs were treated with ECT at the time point and then nuclear and cytosolic fraction were performed. Nrf-2 expression was detected by Western blotting (**A**). Right panel shows the densitometric analysis of Western blot. Immunofluorescence was performed as described in Methods (**B**). Alexa 594-Nrf-2 was detected as red and DAPI was detected as blue. Scale bars = 75 µm, original magnification is 400×. The blots or figure are representative of three independents. * *p* < 0.05, ** *p* < 0.01 vs. control group.

We examined whether ECT regulates the expression of HO-1 and Nrf2 to examine possible defense system against cytokine stimulation. ECT (10–100 μg/mL) upregulated HO-1 expression in a dose-dependent manner; its effect at a concentration of 100 μg/mL ECT similarly appeared that of HO-1 inducer CoPP. HO-1 expression was gradually increased from 4 to 12 h after exposure to ECT (Figure 7). Furthermore, Nrf2 expression in the nucleus was increased by ECT (Figure 8). Nuclear translocation of Nrf2 occurred 2 h after ECT treatment prior to HO-1 upregulation. These data indicate that ECT has a protective effect on vascular inflammation and its action is related to the activation of the HO-1/Nrf2 pathway axis.

## 4. Discussion

Safflower is a medicinal herb that contains a variety of essential pharmacological compounds. Previous studies have reported that its seeds or flowers have numerous components that offer benefits to human health, such as cardiovascular protection, anticoagulant, antioxidant, and hypolipidemic activities, and other metabolic advantages [21]. The present study demonstrated that the ethanol extract of safflower suppresses vascular inflammation via inhibition of the ROS/NF-kB pathway. In addition, safflower was known to regulate the Nrf2/HO-1 axis and exert protective effects against vascular inflammation in HUVECs.

One of the chronic consequences of endothelial dysfunction is the pathological progression to vascular disease. Vascular inflammation is a protective response of tissue that eliminates prolific agents or debris; it is closely related to damage recovery [22]. When endothelial cells undergo inflammatory activation by cytokines, the upregulation of CAMs stimulates the adherence of leukocytes to endothelial cells. In this study, ECT inhibited TNF-α-induced upregulation of ICAM-1, VCAM-1, and E-selectin expression, leading to a decrease in the adhesion of HL-60 monocytes. These results suggest that ECT affects the vascular inflammation process through the inhibition of CAM expression in TNF-α-stimulated HUVECs.

In case of diabetic complication, advanced glycation end products (AGE)/receptor for AGE signaling promotes the production of O_2_^−^, which could further aggravate both the oxidative stress and the impairment of NO [23,24]. Especially, O_2_^−^ is a chemical precursor of many ROS such as H_2_O_2_, and ONOO^−^ in vascular disease. Thus, the overproduction of O_2_^−^ by AGE/RAGE or TNF-α may cause the impaired NO function [25]. TNF-α activates the transcription of proinflammatory gene products through a nuclear translocation of NF-κB and subsequent induction of the CAMs promotor genes. In addition, ROS are involved in all steps of atherosclerosis [26]. ECT treatment decreased the intracellular ROS generation and the nuclear translocation of NF-κB. Furthermore, NF-κB-DNA binding activity was significantly reduced by ECT treatment, suggesting that ECT suppressed vascular endothelial inflammation leading to atherosclerosis via the inhibition of ROS/NF-κB pathway.

Recently, HO-1 expression has been shown to attenuate atherosclerotic processes in vascular endothelial cells, suggesting an anti-atherogenic action [13,27,28]. A human study of HO-1-deficiency showed severe endothelial damage and early atherosclerotic lesions, as reflected by the presence of plaque and fatty streaks [29]. In addition, analysis of polymorphisms in the promoter region of the human HO-1 gene substantiates the beneficial role of HO-1 against atherosclerosis [30]. In the present study, pretreatment with ECT markedly increased Nrf2/HO-1 expression, while suppressing the ROS/NF-κB pathway after TNF-α stimulation. These data demonstrate that ECT regulates the defense system against vascular inflammation, leading to atherosclerosis. Under normal conditions, Nrf2 activity is suppressed in the cytosol by specific binding to the Keap1 chaperone [31].

Electrophilic agents or stimulatory compounds lead to modify thiol groups and then Keap1-mediated repression of Nrf2 activity is lost, then induces Nrf2 nuclear translocation and the potentiation of the ARE response [32]. Finally, this Nrf2/HO-1 induction generate the antioxidant biliverdin and the signaling molecule CO. It is well known that Nrf2/HO-1/CO signaling axis shows protective roles in any type of vascular defense system, and therefore, it might be a potential and useful target as a therapeutic resource for cardiovascular disease.

Drugs that are clinically used to treat cardiovascular diseases should be carefully studied because most of the HO-1 inducers such as hemin or tin compounds have limitations resulting from their cellular toxicity or nephrotoxicity, despite their capabilities [33]. Here, we provide valuable data that ECT shows no cytotoxicity, but only decreases inflammation in vascular endothelial cells.

## 5. Conclusions

**Figure 9:**
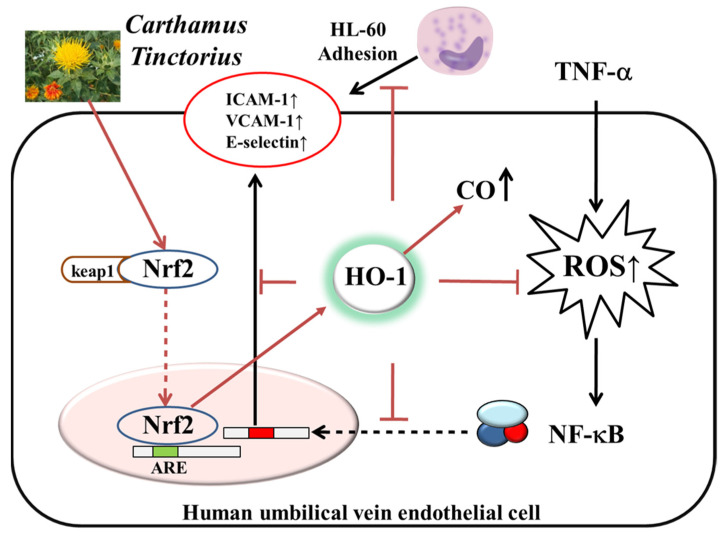
Schematic diagram of the effects of flowers of *C. tinctorius* in vascular inflammation of HUVEC. The potential role of flowers of *C. tinctorius* as a beneficial therapeutic herb inhibited vascular inflammation via ROS/NF-kB pathway. However, modulation of Nrf-2/HO-1/CO signaling axis is involved in its vascular protection in vascular endothelial cells.

In this report, *Carthamus tinctorius* has been shown to have protective effects against vascular inflammation in cytokine-stimulated HUVEC. *Carthamus tinctorius* significantly inhibited expression of cell adhesion molecule, early atherosclerosis marker, via attenuating the ROS/NF-kB pathway. Here, the regulation of Nrf-2/HO-1/CO signaling axis is involved in vascular protection (Figure 9). Therefore, further in vivo studies are required to clarify the therapeutic potential role of *Carthamus tinctorius* to protect vascular disease. In addition, further study will be needed to clarify which compound is dominant for protection of vascular diseases.

## Figures and Tables

**Table 1:** Primers used in PCR.

Gene	Primer Nucleotide Sequence
ICAM-1	Forward: 5′-CTCACCCGTGTACTGGACTC-3′ Reverse: 5′-CGCCGG AAAGCTGTAGATGG-3′
VCAM-1	Forward: 5′-ATGCCTGGG AAGATGGTCGTGA-3′ Reverse: 5′-TGGAGCTGGTAGACCCTCGCTG-3′
E-selectin	Forward: 5′-ATCATCCTGCAACTTCACC-3′ Reverse: 5′-ACACCTCACCAAACCCTTC-3′
MCP-1	Forward: 5’-CAGCCAGATGCAATCAATGC-3’ Reverse: 5’-GTGGTCCATGGAATCCTGAA-3’
β-actin	Forward: 5′-AGGGAGGCGT TCACCTCAGG-3′ Reverse 5′-AACTCCATCACCAGGCG TGGG-3′

**Table 2:** Linear range, regression equation, *r*^2^, LOD, and LOQ for six marker analytes (*n* = 3).

Compound	Linear Range (μg/mL)	Regression Equation (*y* = a*x* + b) ^a^	*r* ^2^	LOD (μg/mL) ^b^	LOQ (μg/mL) ^c^
Quercetin 3-rutinoside	0.31–20.00	*y* = 28,539.15*x* + 2768.62	1.0000	0.06	0.18
Quercetin 3-glycoside	0.31–20.00	*y* = 28,930.08*x* + 1776.94	1.0000	0.04	0.12
Kaempferol 3-rutinoside	0.78–50.00	*y* = 21,078.39*x* + 3828.68	1.0000	0.14	0.43
Hydroxysafflor yellow A	1.56–100.00	*y* = 33,781.29*x* + 3563.25	0.9999	0.19	0.57
Kaempferol 3-glycoside	0.31–20.00	*y* = 38,389.02*x* + 1978.30	1.0000	0.01	0.02
Bidenoside C	0.31–20.00	*y* = 18,983.02*x* + 1748.57	1.0000	0.01	0.04

^a^ *y*: peak area of compounds; *x*: concentration of compounds. ^b^ LOD = 3.3 × *σ*/*S*, ^c^ LOQ = 10 × *σ*/*S* (*σ*: the standard deviation of the *y*-intercept; *S*: the slope of the calibration curve).

**Table 3:** Amount of the six marker analytes in flowers of *C. tinctorius* (*n* = 3).

Compound	Leaves		
	Mean (mg/g)	SD (×10^−2^)	RSD (%)
Quercetin 3-rutinoside	0.02	0.04	2.60
Quercetin 3-glycoside	0.05	0.06	1.25
Kaempferol 3-rutinoside	0.10	0.19	1.81
Hydroxysafflor yellow A	1.59	0.12	0.07
Kaempferol 3-glycoside	0.02	0.06	2.44
Bidenoside C	0.03	0.06	2.33

## Data Availability

The data used to support the findings of this study are included within the article.
